# Association Between Controlled Attenuation Parameter and Mortality in Patients with Nonviral, Nonautoimmune Chronic Liver Disease

**DOI:** 10.3390/diseases14100371

**Published:** 2026-10-07

**Authors:** Mai Totsuka, Ryota Masuzaki, Naoki Matsumoto, Masayuki Honda, Tomohiro Kaneko, Shuhei Arima, Masahiro Ogawa, Hirofumi Kogure

**Affiliations:** Division of Gastroenterology and Hepatology, Department of Medicine, Nihon University School of Medicine, 30-1 Oyaguchi Kami-cho, Itabashi, Tokyo 173-8610, Japan; totsuka.mai@nihon-u.ac.jp (M.T.); matsumoto.naoki@nihon-u.ac.jp (N.M.); honda.masayuki@nihon-u.ac.jp (M.H.); kaneko.tomohiro@nihon-u.ac.jp (T.K.); arima.shuhei73@nihon-u.ac.jp (S.A.); ogawa.masahiro@nihon-u.ac.jp (M.O.); kogure.hirofumi@nihon-u.ac.jp (H.K.)

**Keywords:** controlled attenuation parameter, serum albumin, all-cause mortality, nonviral chronic liver disease

## Abstract

**Background/Objectives:** Controlled attenuation parameter (CAP) is widely used as a noninvasive marker of hepatic steatosis, but its prognostic significance in nonviral chronic liver disease is unclear. We evaluated the association of CAP with all-cause mortality and its relationship with liver stiffness measurement (LSM) and metabolic factors. **Methods:** We retrospectively analyzed 1040 patients with nonviral chronic liver disease who underwent FibroScan assessment in 2022 and were followed through April 2025. Cox proportional hazards models were used to identify factors associated with all-cause mortality. CAP and body mass index (BMI) were evaluated in separate primary multivariable models, with an additional exploratory model including both variables. **Results:** During a median follow-up of 2.8 years, 44 deaths occurred. In a multivariable model adjusted for age, sex, serum albumin, and LSM, lower CAP was independently associated with mortality (HR per 10 dB/m increase, 0.93; 95% CI, 0.89–0.98; *p* = 0.003), together with lower serum albumin and male sex, whereas LSM was not significant. In an exploratory model including both CAP and BMI, the association between CAP and mortality was no longer statistically significant, whereas BMI remained independently associated with mortality. Overall survival differed significantly across CAP quartiles (log-rank *p* < 0.001), with the lowest CAP quartile showing the poorest survival. **Conclusions:** Lower CAP values were associated with increased all-cause mortality; however, this association was no longer statistically significant when BMI was included in the exploratory model. Low CAP should therefore not be interpreted as a favorable phenotype without considering body composition and nutritional status. Further studies incorporating direct measures of sarcopenia and frailty are warranted.

## 1. Introduction

Chronic liver disease remains a major global health burden and is associated with substantial morbidity and mortality worldwide [1,2]. Although hepatocellular carcinoma (HCC) represents one of the most serious complications of chronic liver disease, overall survival is affected by both liver-related and extrahepatic factors, particularly in an aging population [3,4].

In recent decades, the etiologic landscape of chronic liver disease has shifted markedly from viral hepatitis to nonviral causes [5,6,7]. The widespread use of effective antiviral therapies has led to a substantial decline in hepatitis B and C virus–related liver disease, while metabolic dysfunction-associated steatotic liver disease (MASLD) and alcohol-related liver disease have emerged as the predominant etiologies [8,9]. Consequently, nonviral chronic liver disease now represents an increasing proportion of clinical practice.

Patients with nonviral chronic liver disease constitute a clinically heterogeneous population. In addition to liver-specific factors such as fibrosis severity, these patients often present with advanced age, metabolic comorbidities, impaired nutritional status, and frailty, all of which may substantially influence long-term outcomes [10,11]. Therefore, prognostic assessment based solely on the degree of hepatic fibrosis may be insufficient to fully capture determinants of survival, particularly when all-cause mortality is considered.

Transient elastography has become an established noninvasive tool for the assessment of chronic liver disease [12,13]. Liver stiffness measurement (LSM) is widely used as a surrogate marker of hepatic fibrosis, while the controlled attenuation parameter (CAP) provides a quantitative estimate of hepatic steatosis [14,15,16]. Although LSM has been extensively validated as a predictor of liver-related outcomes, the clinical interpretation and prognostic implications of CAP are less clearly defined, particularly with respect to all-cause mortality. CAP is calculated from the attenuation of ultrasonic signals within the liver parenchyma. Because ultrasonic attenuation reflects acoustic scattering and absorption influenced by tissue composition and microstructural heterogeneity, CAP may be affected by factors beyond simple lipid accumulation alone [17]. A low CAP value may not necessarily indicate a favorable clinical condition and may be associated with advanced liver disease, low body mass, impaired nutritional status, sarcopenia, or systemic frailty [18,19]. In this postviral era of metabolic and alcohol-related liver disease, hepatic steatosis should be interpreted in the context of the patient’s overall metabolic and nutritional status.

We therefore hypothesized that CAP may capture systemic and metabolic conditions not fully reflected by fibrosis severity and may provide prognostic information beyond LSM. Accordingly, the present study aimed to evaluate the association between CAP and all-cause mortality, in comparison with LSM, among patients with nonviral chronic liver disease.

## 2. Materials and Methods

### 2.1. Patients

**Figure 1:**
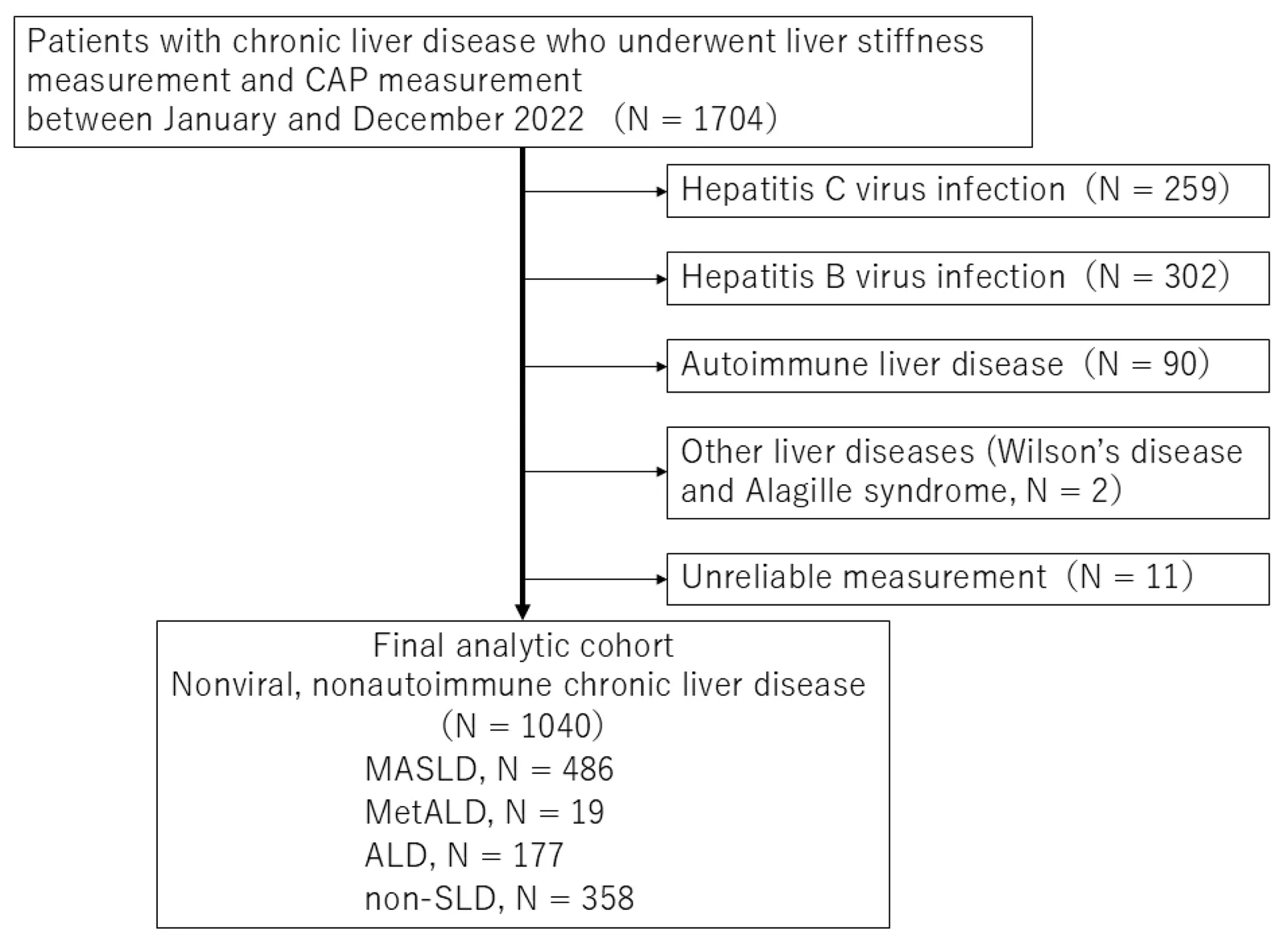
Among 1704 patients with chronic liver disease who underwent FibroScan between January and December 2022, those with viral hepatitis, autoimmune liver disease, other specific etiologies, or unreliable measurements were excluded. A total of 1040 patients with nonviral chronic liver disease were included in the final analysis. The final cohort comprised 486 patients with MASLD, 19 with MetALD, 177 with ALD, and 358 with non-SLD.

This retrospective cohort study included patients aged ≥20 years who underwent liver stiffness measurement (LSM) and controlled attenuation parameter (CAP) assessment using FibroScan (Echosens, Paris, France) at Nihon University Itabashi Hospital between January and December 2022. Among 1704 patients with chronic liver disease, those with hepatitis B virus (HBV) infection, hepatitis C virus (HCV) infection, autoimmune liver diseases, Wilson’s disease, or Alagille syndrome were excluded. Patients with ascites or unreliable FibroScan results, defined as an interquartile range (IQR) to median stiffness ratio >30%, were also excluded. Ultimately, 1040 patients with nonviral chronic liver disease were included in the final analysis (Figure 1).

Demographic, biochemical, and clinical data were extracted from electronic medical records. The etiology of the 1040 patients was further classified as MASLD, MetALD, alcohol-related liver disease (ALD), or non-steatotic liver disease (non-SLD) based on clinical records, alcohol consumption, metabolic risk factors, and evidence of hepatic steatosis. Major metabolic comorbidities, including diabetes mellitus, hypertension, and dyslipidemia, were extracted from the medical records. Heavy alcohol consumption was defined as alcohol intake >60 g/day for men and >50 g/day for women. Baseline laboratory data obtained at the time of FibroScan examination were defined as the start of follow-up. Survival status was ascertained from electronic medical records. Patients without confirmed death were censored at the date of the last hospital visit or 30 April 2025.

The study was conducted in accordance with the Declaration of Helsinki and was approved by the institutional ethics committee of Nihon University Itabashi Hospital. The requirement for written informed consent was waived owing to the retrospective study design.

### 2.2. FibroScan Measurements

Liver stiffness measurement (LSM, expressed in kilopascals [kPa]) and controlled attenuation parameter (CAP, expressed in decibels per meter [dB/m]) were assessed using FibroScan (Echosens, Paris, France) with either the M or XL probe, as appropriate for body habitus. CAP measurements were obtained using a conventional FibroScan system without the SmartExam algorithm or continuous CAP acquisition functionality. All measurements were performed after at least 3 h of fasting.

### 2.3. Outcome Definition

The primary outcome was all-cause mortality. Patients without confirmed death were censored at the date of the last hospital visit or on 30 April 2025, whichever came first.

### 2.4. Statistical Analysis

Continuous variables were expressed as mean ± standard deviation or median (interquartile range), as appropriate, and categorical variables as frequencies and percentages.

For time-to-event analyses, LSM was analyzed both as a continuous variable and as a categorical variable using clinically relevant cut-off values (<8, 8–14.9, and ≥15 kPa), which reflect increasing stages of liver fibrosis. CAP was analyzed as a continuous variable and additionally categorized into quartiles based on its distribution in the study population.

Kaplan–Meier survival curves were generated according to LSM categories and CAP quartiles, and differences between groups were assessed using the log-rank test.

Cox proportional hazards regression analyses were performed to identify factors associated with all-cause mortality. Given the limited number of deaths, clinically selected parsimonious multivariable models were constructed to avoid overfitting. FIB-4 was not included because it incorporates age and laboratory variables and provides information overlapping with LSM. Total cholesterol and LDL cholesterol were not included because they may reflect nutritional and metabolic status overlapping with CAP and BMI.

To avoid simultaneous inclusion of CAP and BMI, which were moderately correlated, two primary multivariable models were constructed. Model 1 included age, sex, serum albumin, LSM, and CAP, whereas Model 2 included age, sex, serum albumin, LSM, and BMI. An additional exploratory model (Model 3) included both CAP and BMI to assess the extent to which the prognostic information conveyed by CAP overlapped with BMI.

As a sensitivity analysis, patients with HCC at baseline or those diagnosed with HCC or an extrahepatic malignancy within 6 months after the baseline examination were excluded, and the multivariable Cox analyses were repeated. CAP was evaluated as a continuous variable in the multivariable models and by quartiles in univariable analysis, with the lowest quartile serving as the reference group. Model discrimination was assessed using Harrell’s concordance index (C-index), with higher values indicating better predictive performance.

To visually explore the joint distribution of age and CAP according to cause of death, a bubble plot was constructed. In this plot, the *x*-axis represents age, the *y*-axis represents CAP, and bubble size corresponds to liver stiffness measurement (LSM, kPa). Horizontal and vertical reference lines indicate CAP = 215 dB/m (the lowest-quartile threshold) and age = 65 years, respectively.

The proportional hazards assumption was assessed using Schoenfeld residuals for the multivariable models. No significant violations were detected for any covariate in Model 3 (global test, *p* = 0.71). Multicollinearity was evaluated using variance inflation factors. Statistical analyses were performed using EZR (Saitama Medical Center, Jichi Medical University), a graphical user interface for R (version 4.0.3). A two-sided *p* value < 0.05 was considered statistically significant.

## 3. Results

### 3.1. Patient Characteristics

A total of 1040 patients with nonviral chronic liver disease were included in the analysis. Of these, 486 patients (46.7%) were classified as MASLD, 19 (1.8%) as MetALD, 177 (17.0%) as ALD, and 358 (34.4%) as non-SLD. Their baseline characteristics are summarized in Table 1. The mean age was 60.7 ± 14.2 years, and 474 (45.6%) were female. The median body mass index (BMI) was 23.9 kg/m^2^ (interquartile range [IQR], 21.3–26.6). The median liver stiffness measurement (LSM) was 5.2 kPa (IQR, 4.0–7.5), and the median controlled attenuation parameter (CAP) was 264 dB/m (IQR, 216–306). The median platelet count was 223 × 10^9^/L (IQR, 181–267), and the median serum albumin level was 4.3 g/dL (IQR, 4.1–4.5).

Regarding alcohol intake, 177 patients (17.0%) consumed >60 g/day (men) or >50 g/day (women). The median FIB-4 index was 1.52 (IQR, 1.04–2.24), and the median eGFR was 70.8 mL/min/1.73 m^2^ (IQR, 60.1–82.2).

Patients in the lowest CAP quartile (≤215 dB/m) were significantly older (median 65 vs. 57 years, *p* < 0.001), had lower BMI (21.1 vs. 26.9 kg/m^2^, *p* < 0.001), lower serum albumin (4.2 vs. 4.4 g/dL, *p* < 0.001), and higher FIB-4 index (1.75 vs. 1.20, *p* < 0.001) compared with the highest quartile (Table 2). Notably, despite having the lowest CAP values, this group had only modestly elevated LSM (median 4.8 kPa), suggesting that low CAP was not primarily driven by advanced fibrosis. Death occurred in 22 patients (8.5%) in the lowest quartile compared with only 2 (0.8%) in the highest quartile (*p* < 0.001).

### 3.2. Survival Analysis

During a median follow-up of 2.8 years, 44 deaths occurred. The estimated 2-year overall survival rate was 96.6%. Twenty patients had hepatocellular carcinoma at baseline, and six additional patients developed hepatocellular carcinoma during follow-up. Causes of death included cardiovascular disease (*n* = 12), extrahepatic malignancies (*n* = 16; pancreatic cancer, *n* = 3; lung cancer, *n* = 3; pharyngeal cancer, *n* = 2; neuroendocrine carcinoma, maxillary gingival cancer, cervical cancer, esophageal cancer, ampullary cancer, bladder cancer, ascending colon cancer, and acute myeloid leukemia, *n* = 1 each), liver-related causes (*n* = 8; liver cancer, *n* = 6; liver failure, *n* = 2), infection (*n* = 5), multiple organ failure (*n* = 1), and other causes (*n* = 2).

### 3.3. Univariable and Multivariable Analyses

In univariable Cox proportional hazards analyses, older age, male sex, lower BMI, lower serum albumin, lower total and LDL cholesterol, higher LSM, lower CAP, higher FIB-4, and lower eGFR were significantly associated with increased all-cause mortality (Table 3). Specifically, older age was associated with a higher mortality risk (HR per 1-year increase, 1.04; 95% CI, 1.02–1.07; *p* = 0.001), and male sex was associated with increased mortality compared with female sex (HR, 2.27; 95% CI, 1.17–4.41; *p* = 0.015). Serum albumin showed a strong inverse association with mortality (HR per 1 g/dL increase, 0.13; 95% CI, 0.08–0.22; *p* < 0.001). Higher LSM was associated with increased mortality (HR per 1 kPa increase, 1.04; 95% CI, 1.02–1.06; *p* < 0.001), whereas higher CAP was associated with lower mortality risk (HR per 10 dB/m increase, 0.93; 95% CI, 0.88–0.98; *p* = 0.003). Diabetes mellitus and heavy alcohol consumption were not significantly associated with mortality.

In multivariable analysis, Model 1 included age, sex, serum albumin, LSM, and CAP. Lower serum albumin, male sex, and lower CAP values remained significantly associated with mortality, whereas LSM was not significant (Table 4).

In Model 2, in which BMI was included instead of CAP, lower BMI, lower serum albumin, male sex, and older age were significantly associated with mortality, whereas LSM remained nonsignificant. The concordance indices were 0.80 for Model 1 and 0.83 for Model 2.

CAP and BMI were moderately correlated (Spearman’s ρ = 0.535, *p* < 0.001), but variance inflation factors were low (maximum, 1.14), indicating no evidence of substantial multicollinearity. In the exploratory Model 3 including both CAP and BMI, CAP was no longer statistically significant, whereas BMI remained independently associated with mortality. The concordance index of Model 3 was 0.83.

A sensitivity analysis excluding 30 patients with HCC at baseline or HCC/extrahepatic malignancy diagnosed within 6 months after baseline yielded results consistent with the primary analysis.

### 3.4. Kaplan–Meier Analysis According to CAP and LSM Categories

**Figure 2:**
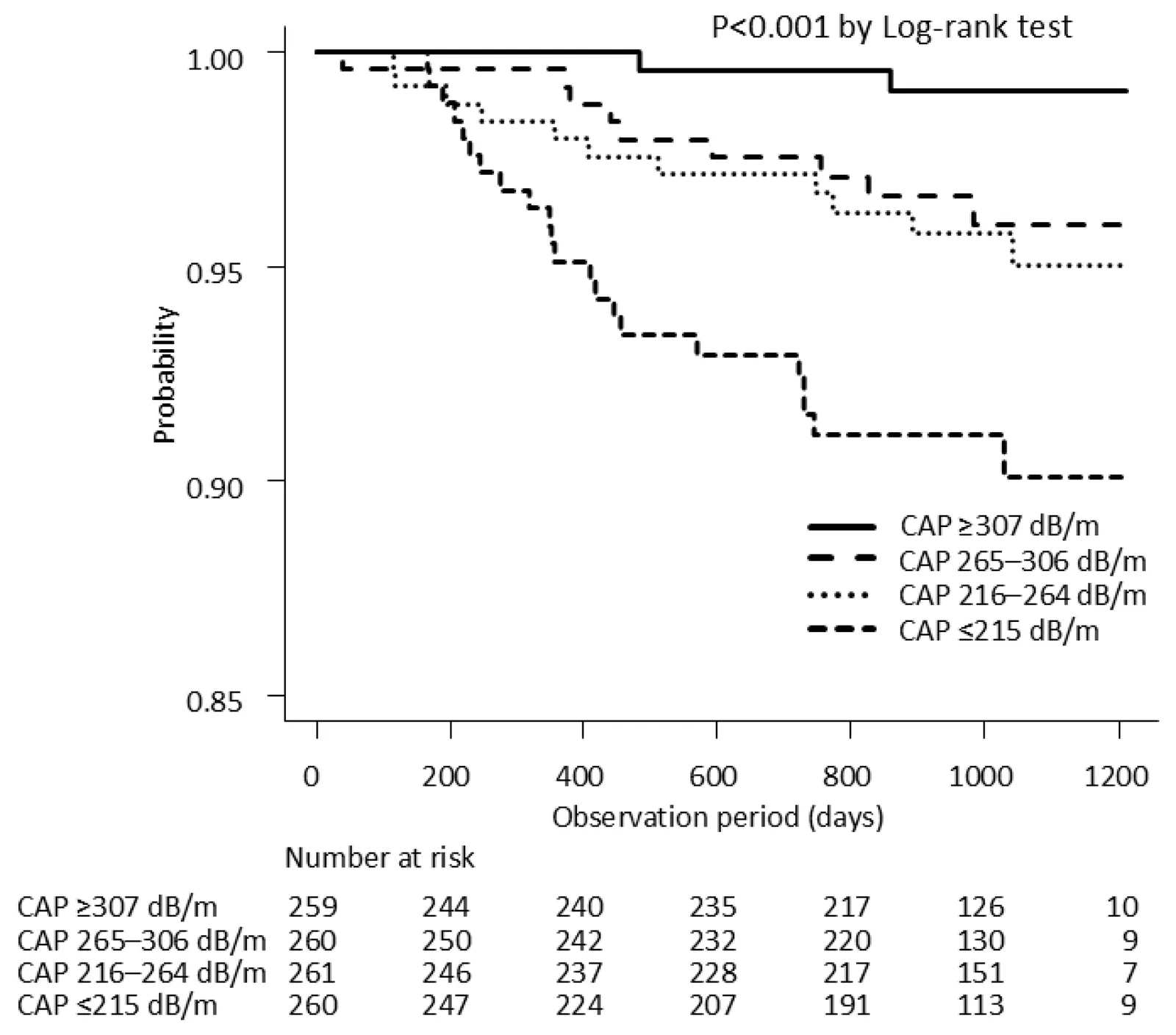
Kaplan–Meier curves for overall survival stratified by quartiles of controlled attenuation parameter (CAP) values (≤215, 216–264, 265–306, and ≥307 dB/m). Overall survival differed significantly among the four groups (log-rank *p* < 0.001). Patients in the lowest CAP quartile (≤215 dB/m) exhibited the poorest survival, whereas those in the highest quartile (≥307 dB/m) showed the most favorable outcomes, demonstrating a stepwise improvement in survival with increasing CAP levels. The number of patients at risk at each time point is shown below the plot. For clarity of visualization, the *y*-axis is truncated from 0.85 to 1.00.

Patients were stratified into quartiles according to CAP values. Kaplan–Meier analysis demonstrated a significant difference in overall survival across CAP quartiles (log-rank *p* < 0.001). Patients in the lowest CAP quartile had the poorest survival, whereas those in the highest quartile had the most favorable outcomes, showing a clear inverse gradient between CAP and mortality (Figure 2).

In univariable Cox analysis using CAP quartiles, higher CAP categories were associated with progressively lower mortality risk compared with CAP ≤ 215 dB/m: HR 0.46 (95% CI, 0.22–0.95; *p* = 0.036) for 216–264, HR 0.38 (95% CI, 0.17–0.82; *p* = 0.014) for 265–306, and HR 0.08 (95% CI, 0.02–0.36; *p* < 0.001) for ≥307 (Table 5).

**Figure 3:**
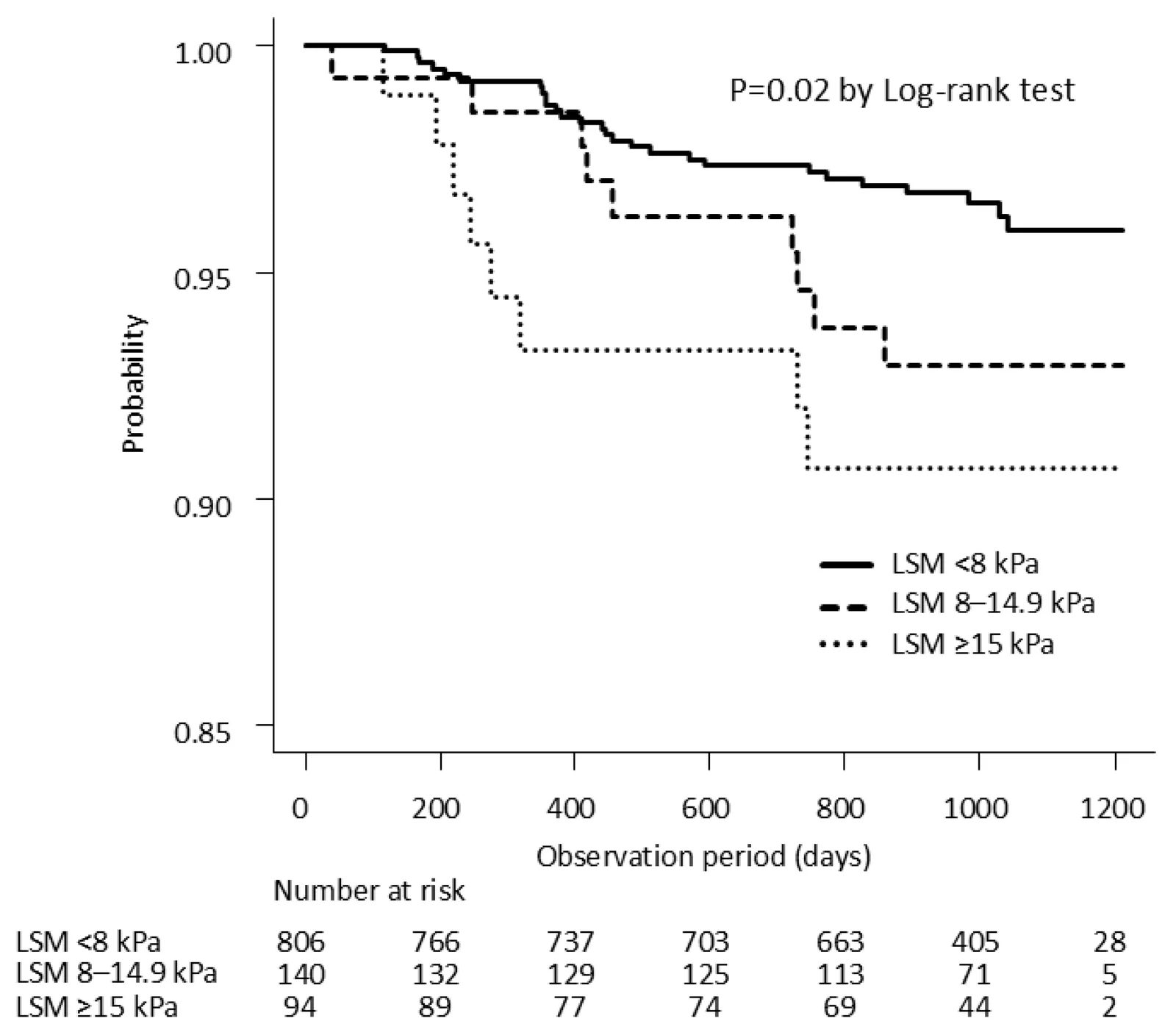
Kaplan–Meier curves for overall survival stratified by liver stiffness measurement (LSM) categories (<8 kPa, 8–14.9 kPa, and ≥15 kPa). Overall survival differed significantly among the three groups (log-rank *p* = 0.02). Patients with LSM ≥ 15 kPa showed poorer survival compared with those with lower LSM values. The number of patients at risk at each time point is shown below the plot. For clarity of visualization, the *y*-axis is truncated from 0.85 to 1.00.

Kaplan–Meier survival curves stratified by LSM categories (<8, 8–14.9, and ≥15 kPa) also showed significant differences in survival (log-rank *p* = 0.02), with poorer outcomes observed in patients with LSM ≥ 15 kPa (Figure 3).

**Figure 4:**
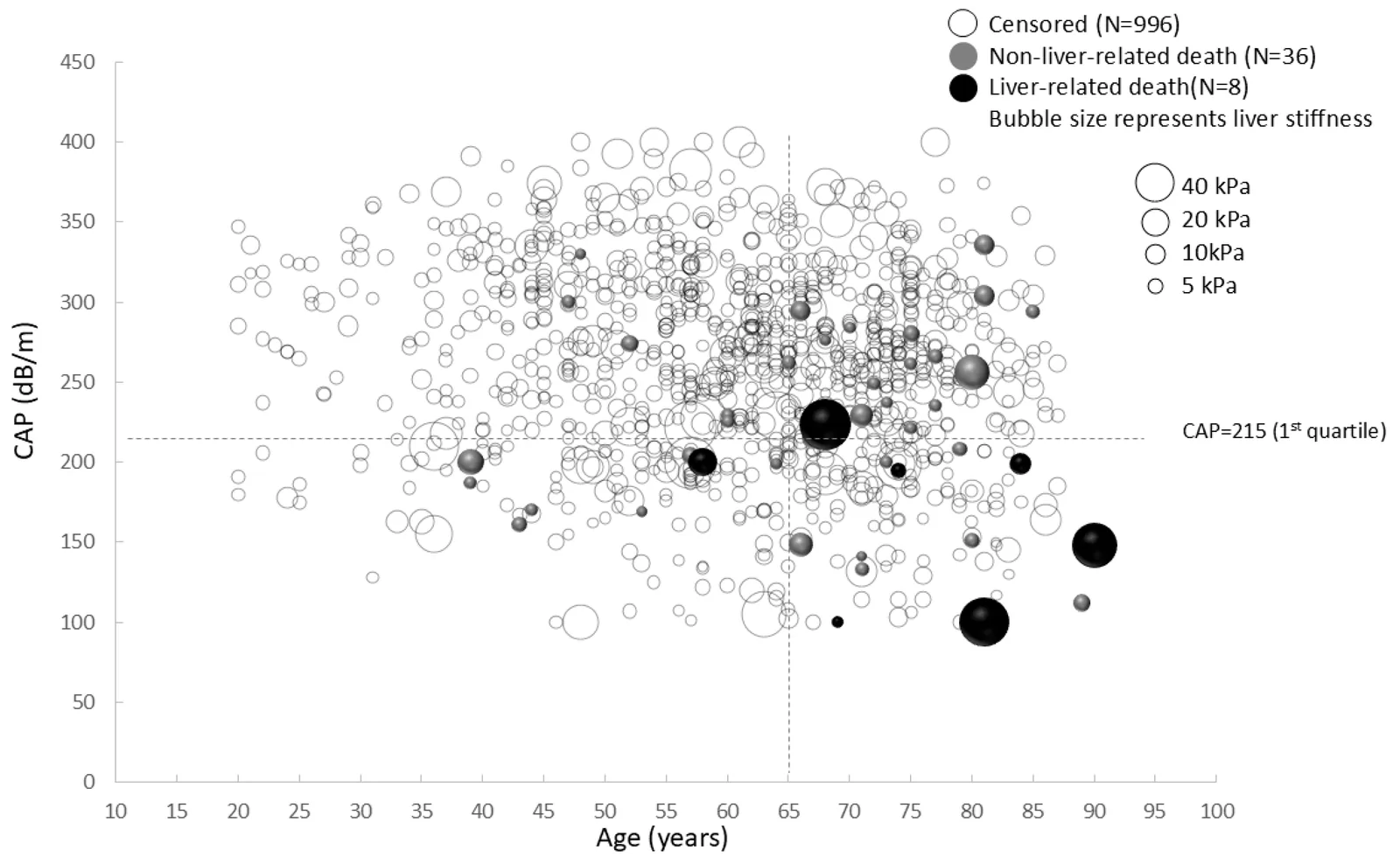
Distribution of age and controlled attenuation parameter (CAP) according to cause of death. Bubble size represents liver stiffness measurement (LSM, kPa). Horizontal and vertical reference lines indicate CAP 215 dB/m (lowest quartile threshold) and age 65 years, respectively. Liver-related deaths clustered among patients with higher LSM values regardless of age or CAP. Non-liver-related deaths occurred predominantly in older patients with lower CAP values, many of whom had relatively low LSM, suggesting that systemic factors rather than advanced fibrosis contributed to mortality in this subgroup.

A bubble plot showing age and CAP distribution by cause of death is shown in Figure 4. Liver-related deaths were predominantly observed in patients with higher LSM values, whereas non-liver-related deaths occurred mainly among older patients with lower CAP values, potentially reflecting impaired nutritional or systemic status. Notably, patients with low CAP values showed a heterogeneous distribution of LSM values.

## 4. Discussion

Previous studies have identified hepatic fibrosis as the most consistent liver-related prognostic factor in patients with nonviral chronic liver disease [20,21,22,23]. However, cardiovascular disease is the principal cause of death in patients with MASLD, and MASLD is associated with an increased risk of fatal and nonfatal major adverse cardiovascular events [24,25]. Extrahepatic malignancies also contribute substantially to long-term mortality in this population [26]. These findings indicate that the prognosis of patients with nonviral chronic liver disease is determined not only by liver disease severity but also by systemic and extrahepatic factors. Metabolic abnormalities may also coexist with other chronic liver diseases and may influence disease progression and the interpretation of CAP. For example, dyslipidemia and hepatic steatosis may coexist in patients with primary biliary cholangitis; however, autoimmune liver diseases, including primary biliary cholangitis, were excluded from the present cohort [27]. Because SLD status, metabolic risk factors, and alcohol consumption may change over time, the etiologic categories used in this study should be regarded as baseline phenotypes rather than fixed classifications.

In this retrospective cohort of patients with nonviral chronic liver disease, lower CAP values were associated with increased all-cause mortality. Although this association persisted after adjustment for age, sex, serum albumin, and LSM, it was no longer statistically significant in the exploratory model including both CAP and BMI. These findings suggest that CAP may capture aspects of body composition and systemic metabolic and nutritional status beyond hepatic fat accumulation alone. Accordingly, hepatic fat content should not be interpreted in isolation from the broader systemic condition of the patient.

CAP is widely used as a noninvasive measure of hepatic steatosis, particularly in MASLD [14,16,28]. In clinical practice, higher CAP values are generally interpreted as indicating more severe steatosis. However, CAP has primarily been validated as a diagnostic marker of hepatic steatosis rather than as a prognostic biomarker. Its ability to quantify hepatic steatosis should therefore be distinguished from its potential association with long-term clinical outcomes.

When CAP and BMI were included simultaneously, CAP was no longer significantly associated with mortality, suggesting substantial overlap with body composition and nutritional or metabolic status. Thus, a lower CAP value does not necessarily indicate a favorable prognosis and may, in some patients, reflect reduced adiposity, sarcopenia, or frailty. Frailty and sarcopenia have been associated with adverse outcomes independently of liver disease severity in patients with advanced liver disease [29,30].

Although the concept of “burned-out” steatotic liver disease, in which hepatic fat content decreases with advancing fibrosis, may account for low CAP in some patients, the median LSM in the lowest CAP quartile was only 4.8 kPa. This suggests that advanced fibrosis was unlikely to be the principal explanation for the low CAP values observed in this cohort. In addition, patients in the lowest CAP quartile had lower BMI and serum albumin levels, suggesting that lower adiposity and impaired nutritional or systemic status may also contribute to low CAP values. Cancer-related malnutrition may also have contributed to low CAP values in some patients, particularly because patients with preexisting malignancies were not systematically excluded. CAP should therefore be interpreted together with BMI and nutritional status rather than as a liver-specific prognostic marker [18,31].

LSM has been repeatedly reported as a strong predictor of liver-related outcomes and mortality in chronic liver disease [12,32,33,34,35]. In the present study, higher LSM values were associated with poorer survival in univariable and Kaplan–Meier analyses but were not significant in the multivariable models. This finding should be interpreted cautiously because serum albumin may partly reflect downstream consequences of advanced liver disease as well as nutritional status. Adjustment for albumin may therefore have attenuated the association between LSM and mortality. Accordingly, the present results do not exclude a prognostic contribution of hepatic fibrosis.

CAP showed a moderate correlation with BMI (Spearman’s ρ = 0.535), and multicollinearity was not evident. Previous biopsy-based studies have demonstrated that BMI independently affects CAP values and that the optimal CAP thresholds for diagnosing hepatic steatosis differ between obese and non-obese patients [36,37]. When CAP and BMI were evaluated in separate multivariable models, both were significantly associated with mortality. However, in an exploratory model including both variables, CAP was no longer statistically significant, whereas BMI remained independently associated with mortality. This finding suggests that the prognostic information conveyed by CAP may overlap with systemic adiposity and metabolic reserve. We do not suggest that CAP is superior to simpler measures such as BMI or serum albumin for assessing nutritional status. Rather, CAP may provide complementary contextual information because it is routinely obtained together with LSM during FibroScan examination. The inverse association between CAP and mortality observed before BMI adjustment is reminiscent of the “obesity paradox” described in chronic diseases, in which greater energy reserves may confer survival advantage under prolonged physiological stress.

These findings suggest that CAP should be interpreted together with LSM and nutritional indicators when assessing mortality risk in patients with nonviral chronic liver disease.

Several limitations should be acknowledged. First, this was a single-center retrospective study with a relatively small number of deaths, which may have limited statistical power. The median follow-up period of 2.8 years was also relatively short and may have preferentially captured mortality related to short-term systemic or nutritional status; longer follow-up is needed to evaluate the long-term prognostic significance of CAP. Second, CAP and LSM were assessed at a single time point, and longitudinal changes were not evaluated. In addition, CAP measurements were obtained using a conventional FibroScan system without the SmartExam algorithm or continuous CAP acquisition, which may affect measurement stability and limit generalizability to newer platforms. In addition, patients with pre-existing malignancies were not systematically excluded, and cancer-related malnutrition may have influenced both CAP values and mortality. The cohort was also heterogeneous with respect to liver disease etiology, which may limit the generalizability of the findings across specific disease subgroups. Finally, detailed assessments of nutritional status and sarcopenia were not available. Further prospective studies are warranted to validate these findings.

Nevertheless, the present findings provide hypothesis-generating insight into the interpretation of CAP in the contemporary metabolic era of chronic liver disease. FibroScan enables simultaneous assessment of liver stiffness and hepatic attenuation in a single noninvasive examination, and CAP may reflect aspects of systemic metabolic reserve in addition to hepatic steatosis.

## 5. Conclusions

Lower CAP values were associated with increased all-cause mortality in patients with chronic liver disease of nonviral etiology. However, this association was no longer statistically significant in the exploratory model including both CAP and BMI. These findings suggest that low CAP should be interpreted in conjunction with BMI and indicators of body composition and nutritional status, and its independent prognostic value remains uncertain. Further studies incorporating direct measures of sarcopenia and frailty are warranted.

## Figures and Tables

**Table 1:** Baseline Characteristics of Patients (*n* = 1040).

Variables	*n* = 1040
Age, years	60.7 ± 14.2
Female, *n* (%)	474 (45.6)
BMI, kg/m^2^ *	23.9 (21.3–26.6)
Serum albumin, g/dL *	4.3 (4.1–4.5)
Total bilirubin, mg/dL *	0.7 (0.5–0.9)
AST, IU/L *	26 (21–37)
ALT, IU/L *	26 (17–41)
T-Chol, mg/dL *	196 (171–224)
LDL, mg/dL *	108 (89–127)
Triglyceride, mg/dL *	114 (80–167)
Platelet count, 10^9^/L *	223 (181–267)
FIB-4 index *	1.52 (1.04–2.24)
LSM, kPa *	5.2 (4.0–7.5)
CAP, dB/m *	264 (216–306)
eGFR, mL/min/1.73 m^2^ *	70.8 (60.1–82.2)
HCC at baseline, *n* (%)	20 (1.9)
Diabetes mellitus, *n* (%)	380 (36.5)
Hypertension, *n* (%)	570 (54.8)
Dyslipidemia, *n* (%)	869 (83.6)
Heavy alcohol consumption, *n* (%) ^†^	177 (17.0)
MASLD/MetALD/ALD/non-SLD, *n* (%)	486 (46.7)/19 (1.8)/177 (17.0)/358 (34.4)

* Values are expressed as median (25th–75th percentiles). ALD, alcohol-related liver disease; ALT, alanine aminotransferase; AST, aspartate aminotransferase; BMI, body mass index; CAP, controlled attenuation parameter; eGFR, estimated glomerular filtration rate; FIB-4, fibrosis-4 index; HCC, hepatocellular carcinoma; LDL, low-density lipoprotein cholesterol; LSM, liver stiffness measurement; MASLD, metabolic dysfunction-associated steatotic liver disease; MetALD, metabolic dysfunction and alcohol-related liver disease; non-SLD, non-steatotic liver disease; SLD, steatotic liver disease; T-Chol, total cholesterol. ^†^ Alcohol consumption >60 g/day for men and >50 g/day for women.

**Table 2:** Characteristics of Patients Stratified by CAP Quartiles (*n* = 1040).

Variables	CAP ≤ 215 (*n* = 260)	CAP 216–264 (*n* = 261)	CAP 265–306 (*n* = 260)	CAP ≥ 307 (*n* = 259)	*p* Value
Age, years	65	64	63	57	<0.001
Female, *n* (%)	147 (56.5)	114 (43.7)	111 (42.7)	102 (39.4)	<0.001
BMI, kg/m^2^	21.1	23.1	25.0	26.9	<0.001
Serum albumin, g/dL	4.20	4.30	4.35	4.40	<0.001
LSM, kPa	4.8	4.9	5.1	6.0	<0.001
Platelet count, 10^9^/L	208	221	233	231	<0.001
FIB-4 index	1.75	1.60	1.40	1.20	<0.001
HCC at baseline, *n* (%)	8 (3.1)	6 (2.3)	4 (1.5)	2 (0.8)	0.244
Deaths, *n* (%)	22 (8.5)	11 (4.2)	9 (3.5)	2 (0.8)	<0.001

Values are presented as median unless otherwise indicated. *p* values were calculated using the Kruskal–Wallis test for continuous variables and Fisher’s exact test for categorical variables. BMI, body mass index; CAP, controlled attenuation parameter; FIB-4, fibrosis-4 index; HCC, hepatocellular carcinoma; LSM, liver stiffness measurement.

**Table 3:** Prognostic Factors for All-Cause Mortality—Univariable Analysis.

Variables	Hazard Ratio (95% CI)	*p* Value
Age (per 1-year increase)	1.04 (1.02–1.07)	0.001
Male	2.27 (1.17–4.41)	0.015
BMI (per 1 kg/m^2^ increase)	0.87 (0.83–0.92)	<0.001
Heavy alcohol consumption ^†^	0.99 (0.44–2.22)	0.98
LSM (per 1.0 kPa increase)	1.04 (1.02–1.06)	<0.001
CAP (per 10 dB/m increase)	0.93 (0.88–0.98)	0.003
Serum albumin (per 1.0 g/dL)	0.13 (0.08–0.22)	<0.001
Total bilirubin (per 1.0 mg/dL)	1.43 (0.89–2.29)	0.14
AST (per 1 IU/L)	1.01 (1.00–1.01)	0.11
ALT (per 1 IU/L)	1.00 (0.99–1.01)	0.86
Platelet count (per 10^9^/L increase)	1.01 (0.97–1.06)	0.49
FIB-4 index (per 1.0)	1.24 (1.12–1.37)	<0.001
T-Chol (per 10 mg/dL)	0.85 (0.79–0.92)	<0.001
LDL (per 10 mg/dL)	0.82 (0.78–0.90)	<0.001
Triglyceride (per 10 mg/dL)	1.00 (0.99–1.00)	0.34
eGFR (per 10 mL/min/1.73 m^2^ increase)	0.82 (0.71–0.96)	0.015
Diabetes mellitus	1.35 (0.75–2.44)	0.32

ALT, alanine aminotransferase; AST, aspartate aminotransferase; BMI, body mass index; CAP, controlled attenuation parameter; eGFR, estimated glomerular filtration rate; FIB-4, fibrosis-4 index; LDL, low-density lipoprotein cholesterol; LSM, liver stiffness measurement; T-Chol, total cholesterol. ^†^ Alcohol consumption >60 g/day for men and >50 g/day for women.

**Table 4:** Prognostic Factors for All-Cause Mortality—Multivariable Analysis.

Variables	Model 1: CAP Model Hazard Ratio (95% CI)	*p* Value	Model 2: BMI Model Hazard Ratio (95% CI)	*p* Value	Model 3: CAP + BMI Model Hazard Ratio (95% CI)	*p* Value
Age (per 1-year increase)	1.02 (1.00–1.05)	0.090	1.03 (1.01–1.06)	0.015	1.03 (1.002–1.06)	0.036
Male	2.61 (1.33–5.13)	0.006	2.91 (1.47–5.76)	0.002	3.01 (1.52–5.99)	0.002
LSM (per 1 kPa increase)	1.01 (0.99–1.03)	0.561	1.01 (0.99–1.03)	0.503	1.01 (0.99–1.03)	0.480
CAP (per 10 dB/m increase)	0.93 (0.89–0.98)	0.003			0.96 (0.91–1.02)	0.157
Serum albumin (per 1 g/dL increase)	0.22 (0.11–0.44)	<0.001	0.22 (0.11–0.42)	<0.001	0.24 (0.12–0.47)	<0.001
BMI (per 1 kg/m^2^ increase)	–	–	0.84 (0.79–0.90)	<0.001	0.86 (0.79–0.92)	<0.001

BMI, body mass index; CAP, controlled attenuation parameter; LSM, liver stiffness measurement. An en dash indicates a variable not included in the model. Number of patients, 1040; number of events, 44; concordance index, 0.80 (Model 1) and 0.83 (Model 2), 0.83 (Model 3).

**Table 5:** Unadjusted Hazard Ratios for All-Cause Mortality According to CAP Quartiles.

Variables	Hazard Ratio (95% CI)	*p* Value
CAP ≤ 215	1.00 (reference)	—
CAP 216–264	0.46 (0.22–0.95)	0.036
CAP 265–306	0.38 (0.17–0.82)	0.014
CAP ≥ 307	0.08 (0.02–0.36)	<0.001

CAP, controlled attenuation parameter.

## Data Availability

The data presented in this study are available on reasonable request from the corresponding author. The data are not publicly available because of privacy and ethical restrictions.

## Abbreviations

The following abbreviations are used in this manuscript:

ALD	alcohol-related liver disease
ALT	alanine aminotransferase
AST	aspartate aminotransferase
BMI	body mass index
CAP	controlled attenuation parameter
CI	confidence interval
eGFR	estimated glomerular filtration rate
FIB-4	fibrosis-4 index
HBV	hepatitis B virus
HCC	hepatocellular carcinoma
HCV	hepatitis C virus
HR	hazard ratio
LDL	low-density lipoprotein cholesterol
LSM	liver stiffness measurement
MASLD	metabolic dysfunction-associated steatotic liver disease
MetALD	metabolic dysfunction and alcohol-related liver disease
non-SLD	non-steatotic liver disease
SLD	steatotic liver disease
T-Chol	total cholesterol
